# Supplementary material for: Symmetry Analysis and Ancestral Sequence Reconstruction Reveal a Symmetrical Translocation Pathway and Activity Determinants of ZIP Metal Transporter
Source: bioRxiv. 2026 May 15:2026.05.13.724914. Preprint. [Version 1] doi: 10.64898/2026.05.13.724914 (PMC13192868; doi:10.64898/2026.05.13.724914)
Supplement: Supplement 1 [file media-1.docx]

Supplementary Information

**Symmetry Analysis and Ancestral Sequence Reconstruction Reveal a Symmetrical Translocation Pathway and Activity Determinants of ZIP Metal Transporter**

Yao Zhang,^1,*^ Tianqi Wang,^1,*^ Hongyan Zhao,^1^ Jian Hu^1,2,‡^

^1^Department of Biochemistry and Molecular Biology, Michigan State University, MI 48824

^2^Department of Chemistry, Michigan State University, MI 48824

^*^Equally contributed to this work.

^‡^Corresponding author: Jian Hu, [hujian1@msu.edu](mailto:hujian1@msu.edu)


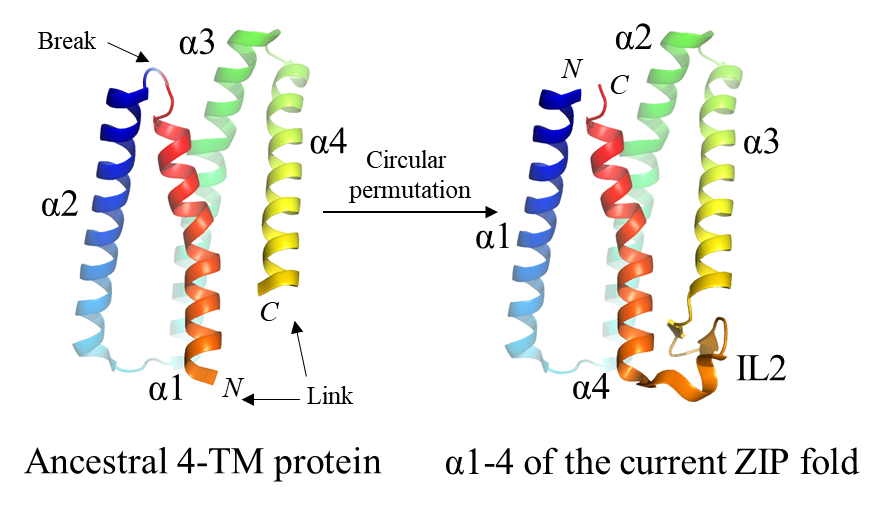


**Figure S1.** Illustration of the proposed circular permutation. TM1-4 in the modern ZIP fold is derived from an ancestral 4-TM protein through circular permutation, which reorders the TMs without affecting the packing of TMs in the 3D structure.

**
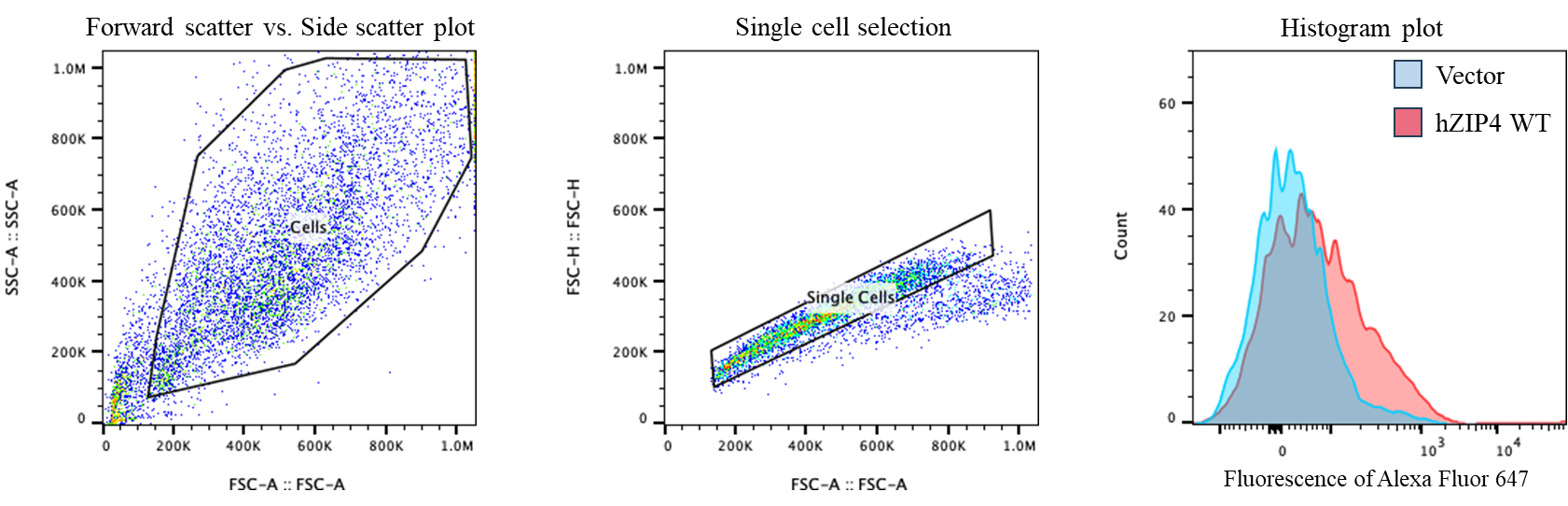
**

**Figure S2.** Flow chart for flow cytometry data processing.

**Table S1.** The primers used in this study.

| Primer | Sequence (forward only, 5’-3’) |
| --- | --- |
| L365A | CACTACATCCTGCAGACCTTCGCGAGCCTGGCAGTGGGTGCACTC |
| L372A | CTGAGCCTGGCAGTGGGTGCAGCGACTGGGGACGCTGTCCTGCAT |
| L380A | ACTGGGGACGCTGTCCTGCATGCGACGCCCAAGGTGCTGGGGCTG |
| L496A | CTGAGCCCAGAGTTGAGGCTAGCGCCCTATATGATCACTCTGGGC |
| M499A | GAGTTGAGGCTACTGCCCTATGCGATCACTCTGGGCGACGCCGTG |
| I500A | TTGAGGCTACTGCCCTATATGGCGACTCTGGGCGACGCCGTGCAC |
| T529A | TCCTGGAAGACCGGGCTGGCCGCGTCGCTGGCCGTGTTCTGCCAC |
| V533A | GGGCTGGCCACCTCGCTGGCCGCGTTCTGCCACGAGTTGCCACAC |
| F597A | CTGGCAGTGGCCACCGGCCTGGCGCTCTACGTAGCACTCTGCGAC |
| V600A | GCCACCGGCCTGTTCCTCTACGCGGCACTCTGCGACATGCTCCCG |
| M605A | CTCTACGTAGCACTCTGCGACGCGCTCCCGGCGATGTTGAAAGTA |
| V533L | GGGCTGGCCACCTCGCTGGCCCTGTTCTGCCACGAGTTGCCACAC |
| D504A | CCCTATATGATCACTCTGGGCGCCGCCGTGCACAACTTCGCCGAC |
| E541A | GCCGTGTTCTGCCACGAGTTGCCACACGCCCTGGGGGACTTCGCCGCCTTGCTGCAC |
| D544A | GAGTTGCCACACGAGCTGGGGGCCTTCGCCGCCTTGCTGCACGCG |
